# Underrecognized pathogen; *Staphylococcus warneri*‐associated native mitral valve endocarditis in an immunocompetent host: A case report and literature review

**DOI:** 10.1002/ccr3.5591

**Published:** 2022-04-22

**Authors:** Mouhammad J. Alawad, Gawahir A. Ali, Wael Goravey

**Affiliations:** ^1^ 36977 Department of internal medicine Hamad Medical Corporation Doha Qatar; ^2^ 36977 Department of Infectious Diseases Communicable Diseases Centre Hamad Medical Corporation Doha Qatar

**Keywords:** coagulase‐negative staphylococci, mitral valve, native valve endocarditis, *Staphylococcus warneri*

## Abstract

*Staphylococcus warneri*‐related endocarditis is rarely reported, raising diagnostic challenges and is often associated with considerable morbidity and mortality. We describe a case of native valve endocarditis caused by *S. warneri* and complicated by a valve perforation in an immunocompetent patient to raise awareness of this emerging organism.

## Graphical abstract

Native valve endocarditis in an immunocompetent host due to *Staphylococcus warneri* is a rare occurrence and often associated with delayed diagnosis. However, vigilance can prevent devastating consequences.

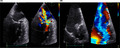

## 1 INTRODUCTION

*Staphylococcus warneri* is a gram‐positive coagulase‐negative Staphylococcus species (CoNS).^1^ It can be isolated in approximately 50% of healthy adults and constitutes around 1% of all skin staphylococci. ^2^ *Staphylococcus warneri* (*S*. *warneri*) is generally considered non‐pathogenic.^2^ However, it constitutes one of the CoNS species sporadically capable of causing human infections. It can present with a variety of infections including bacteremia, line‐related infections, osteomyelitis, and ventriculoperitoneal infections.^3^, ^4^ Immunocompromised patients, elderly individuals, patients with a prolonged hospital stay, and invasive medical devices are usually at risk.^3^

*Staphylococcus warneri* rarely manifests as endocarditis in the native valves, particularly in immunocompetent hosts. The initial clinical presentation and diagnosis are usually indistinguishable between *S*. *warneri*‐related endocarditis and other typical CoNS endocarditis.^5^ Similarly, distinguishing contamination from true bacteremia may be challenging when encountering positive cultures of *S*. *warneri* and can lead to delayed diagnosis and devastating consequences.^1^ Characteristically slow‐progressing infections with subsequent valve destruction are frequently observed if not recognized and promptly treated.^5^

Usually, prolonged antibiotic therapy targeting the organism is the mainstay of management.^6^ Herein, we report an unusual case of *S*. *warneri*‐related endocarditis leading to valve perforation in an otherwise healthy young male patient who was successfully treated with a prolonged course of cefazolin and valve repair. In addition, we reviewed the literature for similar cases.

## 2 CASE PRESENTATION

A 45‐year‐old gentleman presented to the hospital with a 1‐day history of rigor, pleuritic chest pain, and shortness of breath. He reported on and off fever for the past 3 weeks without any other localizing symptoms. His medical history was significant for well‐controlled type 2 diabetes and coronary artery disease for which he is being regularly follow‐up in a specialized heart hospital.

On examination, vital signs showed a fever of 39 °C with normal BP of 125/68 mmHg, heart rate of 108 beats per minute, and saturation of 97% on room air. Chest examination revealed crepitations in the lower‐right zone. No other significant findings were observed. Blood tests were unremarkable except for C reactive protein 55.0 mg/L, urine red blood cells 33 µl, and urine white blood cells 14 µl as depicted in the below Table 1.

**TABLE 1:** Laboratory data

Detail	Value w/Units	Normal range
WBC	16.2 × 10^3^/µl	4–10 × 10^3^/µl
Hb	13 gm/dL	13–17 gm/dl
Platelet	260 × 10^3^/µl	150–400 × 10^3^/µl
Absolute Neutrophil count Auto# (ANC)	14.9 × 10^3^/µl	2–7 × 10^3^/µl
Urea	6.4 mmol/L	2.8–8.1 mmol/L
Creatinine	94 μmol/L	62–106 umol/L
Procalcitonin	0.98 ng/ml	<0.5 negative
CRP	55 mg/L	0–5 mg/L
Urine RBCs	33 µl	0–9 µl
Urine WBCs	14 µl	0–9 µl

His initial blood cultures were negative; however, chest radiography revealed right lower zone infiltrates with no effusion or pneumothorax. Hence, he was admitted and treated for community‐acquired pneumonia with azithromycin and ceftriaxone according to the local hospital policy. Despite this, his fever did not subside, and he developed a new holosystolic murmur in the apex radiating to the axilla on Day 4 of admission. Transthoracic echocardiography revealed mild mitral regurgitation. Subsequently, methicillin‐sensitive *S*. *warneri* was isolated from repeated blood cultures (in one of four blood culture bottles). As a result, the antimicrobial was switched to cefazolin 2 g every 8 h, and transesophageal echocardiography was arranged.

**FIGURE 1:**
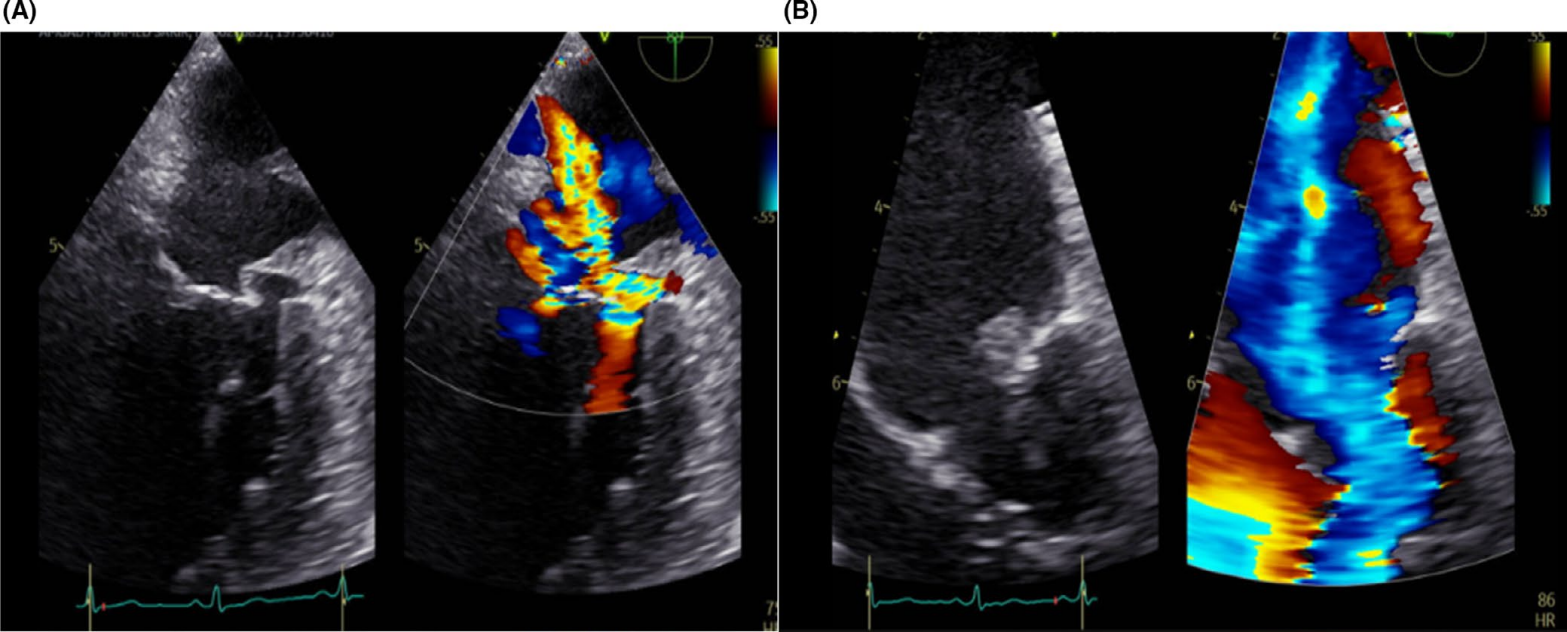
(A and B) Transesophageal echocardiography revealed a perforated posterior mitral valve leaflet with abscess formation and severe mitral valve regurgitation

On Day 6 of admission, transesophageal echocardiography revealed a perforated posterior mitral valve leaflet with an abscess at the level of P1 associated with severe mitral valve regurgitation (Figure 1A,B). On Day 12 after admission, the patient underwent surgical repair of the mitral valve via a superior septal approach. Intraoperatively, perforation in the P1 posterior leaflet of the mitral valve around 0.5 cm with normal commissure was observed. Valve reconstruction using a pericardial patch and mitral ring size 32 mm was performed. Subsequently, methicillin‐sensitive *S*. *warneri* was cultured from the infected valve tissue. The patient had an uneventful postoperative course and was discharged home on Day 14 post‐operatively to continue intravenous cefazolin for a total of 6 weeks through the outpatient antibiotic therapy service. Follow‐up transthoracic echocardiography at 4, 8, and 12 weeks did not reveal any vegetation or mitral valve abnormalities. He has been regularly followed up since then without complications.

## 3 DISCUSSION

Coagulase‐negative staphylococci are commensal human skin and common contaminants in blood cultures.^7^ CoNS is a well‐known cause of prosthetic valve endocarditis (PVE); however, native valve endocarditis (NVE) is increasingly being reported, and 6–7% of NVE is caused by CoNS.^6^ *Staphylococcus epidermidis* followed by *Staphylococcus lugdunensis* has been described as the predominant CoNS causing infective endocarditis in 71.4% and 8.8%, respectively.^8^ *Staphylococcus warneri* has been identified as the causative agent of several invasive infections and rarely endocarditis.^3^ Usually, immunocompromised hosts, recent surgeries and implanted devices are at risk of endocarditis; however, they rarely cause infections in immunocompetent adults.^9^ It is noteworthy, diabetes is associated with increased prevalence and poor outcomes in NVE; whether this is due to increased comorbidities in diabetic patients or a high rate of staphylococcus infections still needs to be identified.^10^ Our patient was young and had no apparent immunosuppressed condition with native heart valves and no history of medical device implantation or recent surgery which makes this case unique. The pathogenesis of CoNS including *S*. *warneri* is determined by it is ability to form biofilms, which is mediated by various proteins and polysaccharide factors, allowing it to escape the immune system and the action of antibiotics. Moreover, recent progress in medicine is associated with the increasing use of medical devices that facilitate infection by CoNS and serve as a nidus for biofilms formation.^11^ Furthermore, *S*. *warneri* can switch from an aggressive form to a dormant and intracellularly adapted lifestyle of infection, eliciting a minimal inflammatory response; thereby facilitating chronic and relapsing infections. This unique phenotypic change is associated with the formation of small colony variants (SCVs) representing naturally occurring and slow‐growing subpopulations.^12^ For these reasons, CoNS, including *S*. *warneri*, has emerged as an important cause of NVE and PVE in the community as well as in healthcare settings; however, further studies are required to delineate the course.^5^ Additionally, the phenomena of formation of SCVs can explain the indolent course and delay diagnosis of endocarditis caused by *S*. *warneri*. As a result, valve complications can occur leading to surgical intervention as in our patient. Furthermore, failure to recognize initial blood culture results as true positives rather than as contaminants might further delay diagnosis and contribute to complications and high mortality despite surgical interventions.^5^, ^6^ However, the distinction between infection and contamination is not always straightforward, and despite many proposed criteria for differentiation but a general and reliable method remains elusive.^1^ In our case, the persistent fever coupled with a new murmur was the hint to consider a positive blood culture of *S*. *warneri* as a true infection; hence, further assessment by echocardiography was performed. The positive culture of *S*. *warneri* from the valve tissue confirmed the initial diagnosis of NVE in this case.

The matrix‐assisted laser desorption ionization (MALDI) TOF MS demonstrates the ability to distinguish *S*. *warneri* from other CoNS because significant interspecies differences exist in clinical relevance, pathogenicity, and antimicrobial susceptibility.^1^ The reported mortality of CoNS NVE, including *S*.* warneri*, is 19%–25% which is higher than that of patients with NVE due to viridans group streptococci.^5^, ^6^

The treatment of *S*.* warneri‐*related endocarditis should be guided by the antimicrobial sensitivity pattern. In contrast to strains acquired in community settings, healthcare‐associated CoNS usually displays a high rate of methicillin resistance (58% vs 22%).^5^ In our case, methicillin‐sensitive *S*.* warneri* was isolated, suggesting community acquisition of infection.

The optimal treatment for *S*. *warneri*‐related endocarditis leading to valve perforation is not yet well defined given it is a rarity. In our case, we treated the patient with a targeted antimicrobial for 6 weeks given the complicated nature of valve involvement.

We searched the PubMed and Google Scholar databases in January 2022 for similar cases. The search terms included “*Staphylococcus warneri*,” “infection,” “bacteremia,” and “Endocarditis.” We excluded infections caused by *S*. *warneri* other than endocarditis and children (<18 years of age). The search was restricted to articles written in English and yielded a total of 14 cases of *S*. *warneri*‐related endocarditis (Table 2). Cases ranged between 28 and 79 years of age and were predominantly male. Only one patient had diabetes,^13^ while almost one‐third had prosthetic valves.^14^, ^15^, ^16^, ^17^ Half of the patients had a recent surgery or device implantation but only one patient described some form of immunosuppressed status. Of the 14 cases reviewed, five reported symptoms more than 2 weeks, although the data were not obtainable in 4 cases.^3^, ^4^, ^18^, ^19^ Aortic valves were mostly involved, and in almost one‐fifth of the cases, both the aortic and mitral valves were involved.^3^, ^13^, ^20^ Of the cases identified, methicillin‐sensitive *S*.* warneri* was isolated in almost half as in our case.^4^, ^9^, ^13^, ^18^, ^21^, ^22^ The duration of therapy ranged from 4 to 8 weeks, although data were not always available.^19^, ^23^ Only one death was identified in our review though 43% of the cases necessitated some form of surgical procedure, whether replacement or debridement while 5 cases developed metastatic manifestation of endocarditis (Table 2).

**TABLE 2:** Summary of previously reported adult cases of *S. warneri* endocarditis

	Case	Sex/age	Comorbidities	Prosthetic valve	Immunosuppressive conditions	Recent surgery or devices implantation	Duration of the symptoms	Valve involved	Valve surgery	Metastatic manifestations	Definite Antibiotics and duration	Outcomes
1	Dan M et al, 1984^21^	M/32	Nil	No	No	Yes	2 weeks	AV	Yes	No	Penicillin, 4 weeks	Alive, prosthetic valve
2	Wood et al, 1989^20^	M/66	HTN, OA, hip prosthesis	No	No	Yes	>6 weeks	AV & MV	Yes	Yes	Vancomycin, gentamicin, rifampicin, 6 weeks	Alive, surgical replacement of MV and debridement of AV
3	Kamath et al, 1992^3^	M/64	Liver cirrhosis	No	No	No	NA	AV & MV	No	Yes	Vancomycin, gentamicin, 14 days	Death
4	Abgrall et al, 2001^14^	M/71	Mechanic prosthetic AV	Yes	No	Yes	5 days	AV	Yes	No	Vancomycin, Pefloxacine, 6 weeks	Alive, bioprosthetic valve
5	Stöllberger et al, 2006^23^	M/48	Disc prosthesis in L 4/5	No	No	Yes	>6 months	AV	No	No	Vancomycin, fucidic acid oral, Rifampicin, no define duration	Alive
6	Kini et al, 2010^4^	F/78	Nil	No	No	No	NA	MV	No	No	Nafacilin, 6 weeks	Alive
7	Arslan et al, 2011^15^	F/43	Congenital AS, IE	Yes	No	Yes	20 days	AV	No	Yes	Ampicillin/sulbactam, gentamicin, 8 weeks	Alive
8	Bhardwaj et al, 2016^22^	M/59	Nil	No	No	No	3 days	MV	No	No	Cefazolin, 6 weeks	alive
9	Kuvhenguhwa et al, 2017^16^	M/67	Bioprosthetic aortic, CVA, CABG	Yes	No	Yes	1 week	AV	No	No	Vancomycin, rifampicin, 6 weeks	Alive
10	Diaconu et al, 2019^18^	M/79	HTN	No	No	No	NA	MV	No	No	Oxacillin, 1 month	Alive
11	Yamamoto et al, 2020^13^	M/49	DM	No	No	No	8 days	AV & MV	Yes	No	Cefazolin, 4 weeks	Alive, prosthetic valves
12	Sunderland et al, 2021^17^	M/64	Bioprosthetic aortic, CABG	Yes	Yes, Nephrotic syndrome	Yes	10 days	AV	Yes	No	gentamicin, vancomycin, rifampicin, 28 days	Alive, surgical replacement AV
13	Kurihara et al, 2021^9^	F/72	MR	No	No	No	3 weeks	MV	No	Yes	Penicillin G, 6 weeks	Alive
14	El Nakadi et al, 2021^19^	F/28	Nil	No	No	No	NA	AV	Yes	Yes	NA	NA
15	Our case	M/45	DM, CAD	No	No	No	3 weeks	MV	Yes	No	Cefazolin, 6 weeks	Alive, Valve repair

Abbreviations: AS, Aortic stenosis; AV, Aortic valve; CABG, Coronary artery bypass graft; CAD, Coronary artery disease; CVA, Cerebrovascular accident; DM, Diabetes mellitus; HTN, Hypertension; IE, Infective Endocarditis; MR, Mitral regurgitation; MV, Mitral valve; NA, Not available; OA, Osteoarthritis.

## 4 CONCLUSION

*Staphylococcus warneri* native valve endocarditis in an immunocompromised host is a rare clinical entity and even rarer in an immunocompetent host that demonstrates the ability of the organism to manifest as an invasive infection. Therefore, careful clinical judgment is needed when encountering *Staphylococcus warneri* in the blood to differentiate true infection from contamination to avoid valve destruction and detrimental consequences. Treatment should be guided by the pattern of antimicrobial sensitivity and the optimal treatment duration remains unknown, but 4–8 weeks is suggested.

## CONFLICT OF INTEREST

The authors declare that they have no competing interests.

## AUTHORS CONTRIBUTIONS

MA involved in clinical management, data acquisition, literature search, and original manuscript writing. GA involved in clinical management, literature search, and manuscript writing. WG supervised all the aspects and contributed to final manuscript editing and proof reading.

## ETHICAL APPROVAL

Ethics approval and permission was obtained to publish the case reports from the institutional review board which is in line with international standards,

## CONSENT

Written informed consent was obtained from the patient to publish this report in accordance with the journal’s patient consent policy.

## Data Availability

The authors confirm that the datasets supporting the findings of this case are available from the corresponding author upon request.
